# Detection of natural autoimmunity to ghrelin in diabetes mellitus

**DOI:** 10.3389/fmedt.2024.1407409

**Published:** 2024-07-12

**Authors:** Rega H. Kasim, Thilo Samson Chillon, Anna Maria Eleftheriadou, Eddy Rijntjes, Waldemar B. Minich, Stefan Zechmann, Lutz Schomburg

**Affiliations:** ^1^ Institute for Experimental Endocrinology, Charité—Universitätsmedizin Berlin, Berlin, Germany; ^2^ Division of Diabetes and Endocrinology, GZO Zurich Regional Health Center, Wetzikon, Switzerland

**Keywords:** diabetes mellitus, autoantibody, autoimmune disease, *in vitro* diagnostics, sexual dimorphism, growth hormone

## Abstract

**Objective:**

Ghrelin is an orexigenic peptide that becomes post-translationally modified. Natural autoantibodies to ghrelin (ghrelin-aAb) have been described in healthy subjects, in eating disorders and rheumatic diseases, with potential clinical relevance. Despite these important reports, the data base on the prevalence and physiological role is small and technical approaches for assessing ghrelin-aAb are few, encouraging respective research for improving knowledge on the potential endocrine significance.

**Methods:**

A novel immunoprecipitation assay was generated based on a fusion protein of human ghrelin with a reporter gene. Assay quality was verified with commercial antibodies. Assay characteristics and matrix effects were determined, including stability of natural ghrelin-aAb to freezing, signal linearity in dilution experiments, and comparison of different matrices. Three groups of serum samples were analyzed for ghrelin-aAb, comprising commercial sera from healthy subjects and patients with type 1 or type 2 diabetes mellitus.

**Results:**

The newly generated ghrelin-aAb assay proved sensitive, robust and reliable over a broad concentration range. Results from serum and plasma differed slightly. The signals from serum remained stable towards freezing and thawing, and in dilution experiments. Applying a mathematical criterion for outliers (P75 + 1.5-times IQR), an average prevalence of 11%–12% of positive samples was identified in the different human cohorts, with no significant sex-or disease-related difference.

**General significance:**

A novel diagnostic autoantibody assay detected ghrelin-aAb with a similar prevalence in diabetic patients and controls, suggesting that autoimmunity to ghrelin plays little role in diabetes mellitus, but may be of relevance in other diseases where ghrelin signaling is essential.

## 1 Introduction

The arcuate hypothalamic nucleus (ARC) acts as the appetite regulatory center in the human body. It is part of the hypothalamus and is adjacent to the third ventricle and the median eminence. In response to specific stimuli such as food intake or fasting, peripheral organs and tissues in the body release appetite-regulating hormones as humoral signals to the neuron population of the ARC to maintain homeostasis (1, 2). The gut-derived peptide ghrelin elicits positive appetite-inducing effects, acting as an endogenous orexigenic signal secreted mainly before meal intake (3). Furthermore, it has been described as a modifier of glucose- and energy-homeostasis, heart and muscle protection, and also as being involved in bone metabolism and tumorigenesis (4). The endocrine and orexigenic effects of ghrelin are mainly mediated by the growth hormone secretagogue receptor (GHS-R), found e.g., on NPY/AgRP neurons in the ARC and on somatotropic cells of the anterior pituitary (5).

Since its detection, ghrelin has been increasingly associated with obesity and insulin resistance in mammals, controlling energy homeostasis, growth hormone release and lipogenesis (6). Subcutaneous injections of ghrelin caused weight gain in rodents by increasing food intake, insulin secretion and reducing fat utilization (7). In human subjects, low ghrelin plasma concentration were associated with circulating insulin concentrations, hypertension, and type 2 diabetes mellitus (T2DM) (8). These findings amongst others highlight the potential relevance of ghrelin for controlling metabolism as a novel pharmacological target for obesity, insulin resistance, fatty liver disease and T2DM (9, 10).

It is therefore conceivable that an imbalance in ghrelin signaling may influence metabolic disease risks and effect energy metabolism, with pathophysiological relevance (11). Accordingly, gene variants have been described, and polymorphisms in the ghrelin gene were associated with hypertension in T2DM (12), and alcohol use disorder (13).

Besides genetic effects, autoimmunity to ghrelin signaling has been described recently, in particular concerning obesity (14), eating disorders (15–18) and rheumatoid arthritis (19). Mechanistically, the endogenous ghrelin-aAb may stabilize circulating ghrelin, thereby increasing its half-life and supporting its endocrine signaling activity (20). Alternatively, the interaction of ghrelin with its receptors may become impaired, inducing ghrelin resistance.

By now, detection of natural ghrelin-aAb in human serum samples has mainly been performed by enzyme-linked immunosorbent assays (ELISA) using commercial ghrelin preparations as bait, and employing anti-human IgG with reporter moiety as detectors for recognizing the endogenous ghrelin-aAb (14, 21). As the different techniques come with specific advantages and limitations, we decided to establish an alternative method for ghrelin-aAb quantification by using a fusion protein of ghrelin in frame with a reporter enzyme as bait, thereby avoiding a second detection step. To this end, protein-A-based precipitation of potential aAb bound to the fusion protein is used for quantification of ghrelin-aAb. After verification of the suitability and robustness of the method, we decided to conduct a first analysis of control subjects in comparison to patients with a diagnosis of T1DM or T2DM. The results indicate high quality of the analytical assay, but no differences in ghrelin-aAb prevalence in the three groups of subjects.

## 2 Material and methods

### 2.1 Human serum samples

Three sets of serum samples obtained from healthy human adults and patients with a diagnosis of T1DM or T2DM were purchased from a commercial supplier (in.vent Diagnostica GmbH, Hennigsdorf, Germany). The anthropometric and pathophysiological information available for these samples was restricted to the diagnosis of T1DM or T2DM, or a self-reported health status as disease-free (“healthy”), along with age and sex at the time of blood drawing. The full cohort available for analysis consisted of healthy subjects (200 males and 200 females, age range; 18–63 years), 121 patients with T1DM (60 males and 61 females, age range; 18–78) and 124 patients with T2DM (66 males and 58 females, age range; 28–87). Additional clinical information was not available due to data safety regulations. Ethical permission and written informed consent of all subjects analyzed in this study were collected by the commercial provider prior to blood drawing, aliquot preparation and commercial distribution to the research laboratory.

### 2.2 Materials

A commercial polyclonal IgG antiserum to human ghrelin was purchased (#PA1-1046, Invitrogen, Thermo Fisher Scientific GmbH, Deutschland,) to serve as positive control. The antiserum is described to detect both the octanoylated and non-octanoylated forms of ghrelin. 96-well plates were obtained from Greiner AG (Kremsmünster, Austria), and SEAP substrate Tropix CSPD was purchased from Applied Biosystems GmbH (Darmstadt, Germany).

### 2.3 Construction and preparation of the ghrelin-SEAP fusion reporter

The cDNA of secreted alkaline phosphatase (SEAP) was amplified by PCR and inserted into plasmid pIRESneo giving rise to pIRESneo-SEAP, as described before in the generation of a similarly-designed assay in more detail (22). The cDNA of human ghrelin was amplified by PCR and used to generate plasmid pIRESneo-SEAP-ghrelin encoding the SEAP-ghrelin fusion protein. The expression plasmids were verified by DNA sequencing. Human embryonic kidney cells (HEK 293 cells) were grown in DMEM/F12 (#31330, Thermo Fisher Scientific GmbH, Dreieich, Germany) supplemented with 10% fetal bovine serum and transfected with pIRESneo-SEAP-ghrelin using FuGENE HD reagent (#E2311, Promega GmbH, Walldorf, Germany). Forty-eight hours after transfection, the selection of stable transfectants was started by adding 0.8 mg/ml G418 sulfate (#345812, Calbiochem GmbH, Sandhausen, Germany). Stable clones expressing high levels of recombinant protein were selected and expanded for protein production. To this end, confluent HEK 293-SEAP-ghrelin cells were grown in 75 cm^2^ plates in serum-free DMEM/F12 medium containing 1% BSA for 72 h. The cell culture supernatant was collected, centrifuged at 2.500 rpm to pellet cells and large debris, decanted, and stored at −80°C until needed for the measurements.

### 2.4 Immunoprecipitation assay for ghrelin-aAb using human sera

The cell extract containing SEAP-ghrelin was diluted in assay buffer (20 mM HEPES-NaOH, pH 7.5, 50 mM NaCl, 1% Triton X-100, 10% glycerol, and 5 mg/ml BSA). Measurements were conducted with 40 μl SEAP-ghrelin-buffer dilution and 5 μl of serum sample, and incubated overnight at 4°C. The next day, 40 μl of 20% POROS-Protein-A resins (ASKA Biotech GmbH, Hennigsdorf, Germany) were added and incubated for 1 h at room temperature. POROS-Protein-A-immune complexes were precipitated and washed 6 times with 200 µl of washing buffer (50 mM Tris-HCl, pH 7.5, 100 mM NaCl, 0.5% Triton ×100). Washed pellets were dissolved in 150 µl SEAP substrate buffer (Tropix CSPD, Applied Biosystems) pre-diluted 1:5 in substrate buffer (1M diethanolamine, 0.5 mM MgCl2), and incubated for 30 min. SEAP activity was measured in a luminometer for 5 s and the relative light units (RLU) were recorded. Results are expressed as RLU, or after normalization to the background as a binding index (BI), denoting the signal strength as factor above average control signals.

### 2.5 Isolation of human immunoglobulins

In order to isolate human immunoglobulins (Ig), serum samples (100 µl) were diluted with assay buffer to 1.0 ml and incubated overnight at 4°C with 0.2 ml of protein G-Sepharose known to bind Ig, mainly of the IgG class. Complexes were pelleted and washed 10 times with PBS. Bound Ig were eluted with 25 mM citric acid (pH 3.0), and immediately neutralized to a pH of 7.0 using 1M HEPES-NaOH (pH 8.0). Eluted Ig were concentrated to 100 μl (∼1 mg/ml) using a Speedvac device at room temperature.

### 2.6 Statistical analysis

GraphPad Prism 8 software was used to analyze the data. All data are represented as mean ± S.E.M. Statistical significance was defined as *p* < 0.05 (*), *p* < 0.01 (**) or *p* < 0.001 (***). Signals were normalized according to the background noise from negative samples. To this end, the mean of the signals (RLU; relative light units) from the lower 50% of samples analyzed was determined, and received a binding index (BI) of 1.0. Then, all signals were divided by this background RLU value, thereby converting the measured RLU to BI values. Using a mathematical outlier criterion (P75 + 1.5-times IQR), a threshold for positivity was defined from the full set of BI values, assuming that most samples are negative for ghrelin-aAb. Samples were classified as positive or negative for ghrelin-aAb when the calculated BI exceeded this threshold value or not, as described (23). This definition is robust for medium-sized cohorts, and applicable to data sets containing aAb-positive samples (i.e., mathematical outliers), under the condition that the number of aAb-positive samples is less than 25% of all samples tested, which can be safely assumed in observational cross-sectional studies.

## 3 Results

### 3.1 Generation of an immunoprecipitation assay to measure ghrelin-aAb

**Figure 1:**
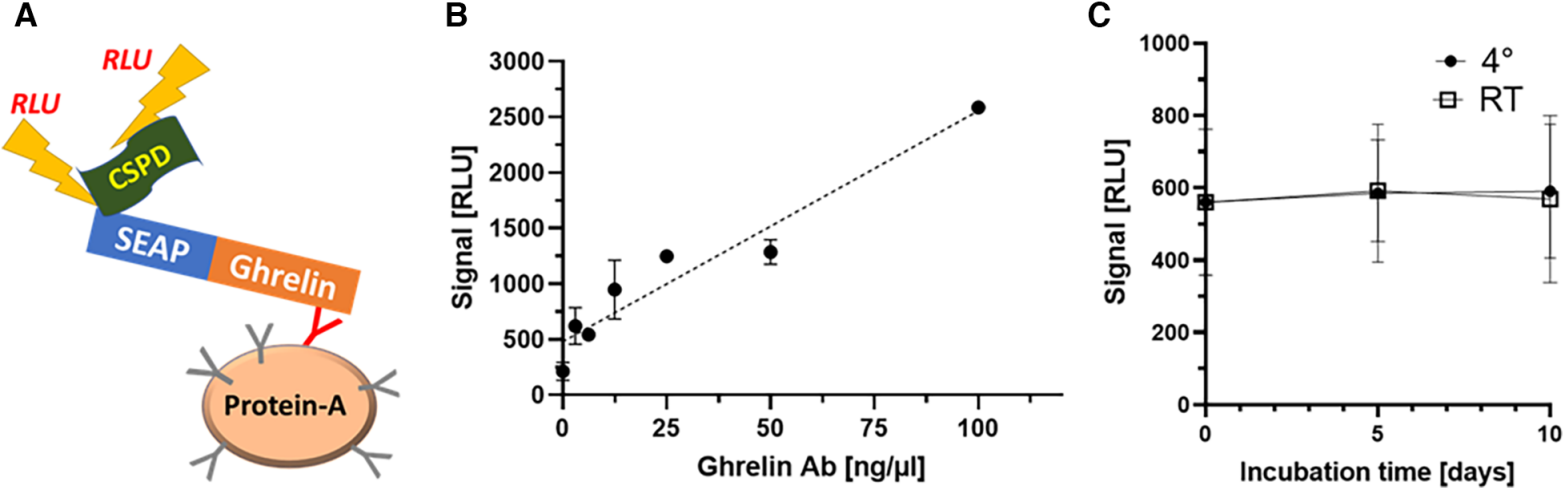
Establishment and verification of the new method and characterization of stability of ghrelin-aAb in serum. (**A**) A detection assay was designed, consisting of a secreted alkaline phosphatase (SEAP) in frame to human ghrelin as one fusion protein, to be used as bait for the autoantibodies (Y-symbol, ghrelin-specific immunoglobulin highlighted in red) that become enriched by protein A-mediated immunoprecipitation. Detection is achieved by incubation with a chemiluminescent substrate (CSPD), and relative light units (RLU) are recorded by a luminometer. After the establishment of stable clones expressing SEAP-ghrelin fusion protein, the signal detection method and certain matrix characteristics were characterized. (**B**) Increasing signal strength (RLU) was observed with increasing amount of commercial anti-ghrelin antibody in the new assay, supporting its general suitability to detect and quantify ghrelin-aAb. (**B**) Signal stability of positive samples was tested by incubation for 5 days and 10 days at room temperature or 4°C. The comparison to the initial signal strength indicates high stability of ghrelin-aAb in serum under these conditions.

After transfection and G418-mediated selection, a total of twelve HEK293 cell clones were isolated and expanded. Expression levels of the SEAP-ghrelin fusion proteins were determined by assessment of SEAP enzymatic activity. The two clones with the highest SEAP activity were selected, further expanded and stocks were prepared for safe storage in liquid nitrogen. A subset of the sera from the healthy subjects was analyzed to identify samples with positive ghrelin-aAb to serve as regular standards and for test performance characterization. A commercial antiserum recognizing human ghrelin was tested in parallel to validate the assay and assess signal dependence on Ab concentration (Figure 1A). Two positive samples were tested for stability at 4°C or room temperature (RT) for 5 and 10 days. The results indicate no loss of ghrelin-aAb signals in serum under these conditions (Figure 1B).

### 3.2 Characterization of signal strength with varying amounts of ghrelin-aAb

**Figure 2:**
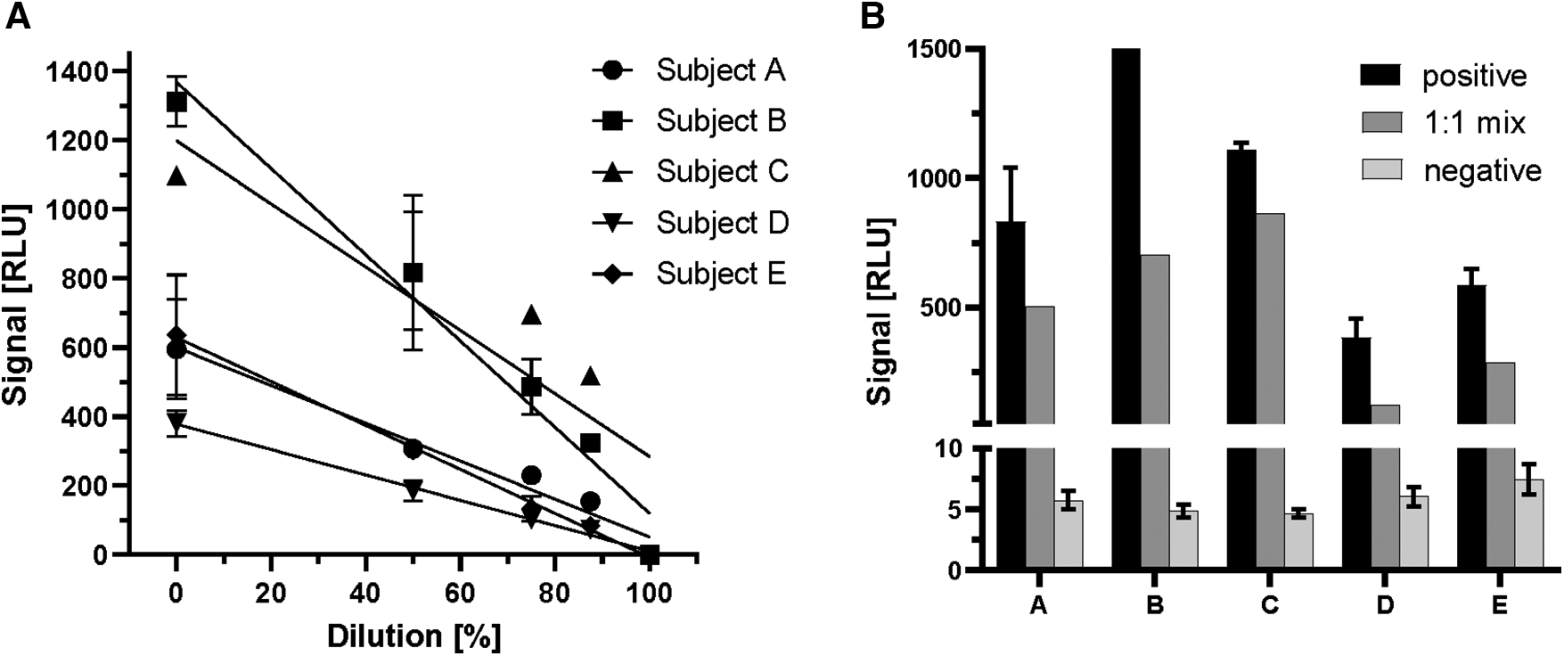
Characterization of ghrelin-aAb signal decline with dilution. Signal strength was tested with positive serum samples in dilution experiments with assay buffer or negative serum samples. (**A**) Five positive serum samples (subjects A–E) were diluted with assay buffer to 50%, 25%, 12.5% and 0.1% of the original composition. The signals declined steadily with increasing dilution. (**B**) Five different ghrelin-aAb positive serum samples were diluted 1:1 with a negative serum sample each. Signal strength for ghrelin-aAb declined strongly, towards the arithmetic mean of both samples, indicating little to no matrix effects.

After verifying the proportional increase of signal strength with ghrelin-specific commercial antiserum to human ghrelin, serum samples positive for endogenous ghrelin-aAb were tested in dilution experiments. To this end, linear dilution of positive samples with assay buffer was tested over a wide concentration range (Figure 2). The samples displayed the expected decrease in signal strength with dilution (Figure 2A). Next, equi-volume mixtures of a positive and a negative serum were prepared and signal strength for ghrelin-aAb was determined. The results indicate little matrix effects, with the majority of mixtures yielding a signal close to the calculated arithmetic mean of the positive and negative signal strengths (Figure 2B).

### 3.3 Comparison of different matrices and stability towards freezing

**Figure 3:**
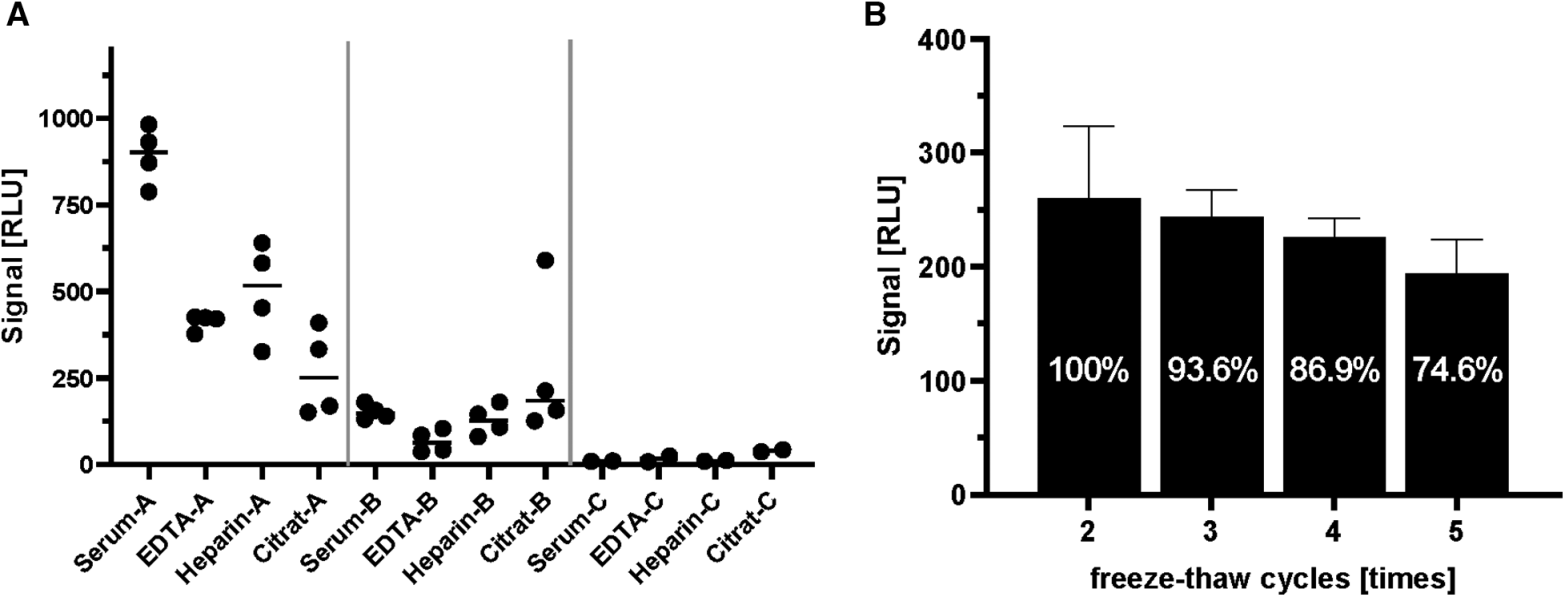
Comparison of serum and plasma matrices and effects of freezing on ghrelin-aAb signal strength. Different matrices from human blood and effects from repeated freezing were compared with respect to ghrelin-aAb signals, taking both total signal strength and variation of the measurement into account. (**A**) Signals varied when ghrelin-aAb signals from serum were compared with matched plasma preparations containing EDTA, heparin or citrate as an anticoagulant. Serum and EDTA-plasma turned out to generate the most stable signals, with serum yielding higher RLU and thus qualifying as the preferential matrix for ghrelin-aAb analysis. (**B**) Signal strength declined upon multiple cycles of freezing and thawing, but ghrelin-aAb were still detected with more than 85% of the original signal strength after 4 freeze-thaw cycles.

In large clinical studies, different sample matrices (serum/plasma) are used for the analysis of laboratory parameters. For this reason, samples prepared by different methods of sample collection (serum, EDTA as anticoagulant, heparin as anticoagulant, citrate as anticoagulant) from two positive and a negative individual were compared side-by-side to characterize the suitability of different matrices for the ghrelin-aAb assay (Figure 3). Inhomogeneous results were obtained, with serum yielding the highest RLU from a positive donor (Figure 3A). The analysis of large-scale observational and intervention studies from stored samples often involves several freezing and thawing steps, before a specific analytical measurement can be conducted. Samples with one or no previous freezing cycle are rarely available, and were thus not included in the analysis. In order to test the stability of the ghrelin-aAb signals towards repeated freezing, a positive sample underwent a series of freeze-thaw cycles. Signal intensity was calculated as percentage of the maximum. The results indicate a gradual but moderate signal loss under these conditions (Figure 3B).

### 3.4 Ghrelin-aAb prevalence in healthy subjects and diabetic patients

**Figure 4:**
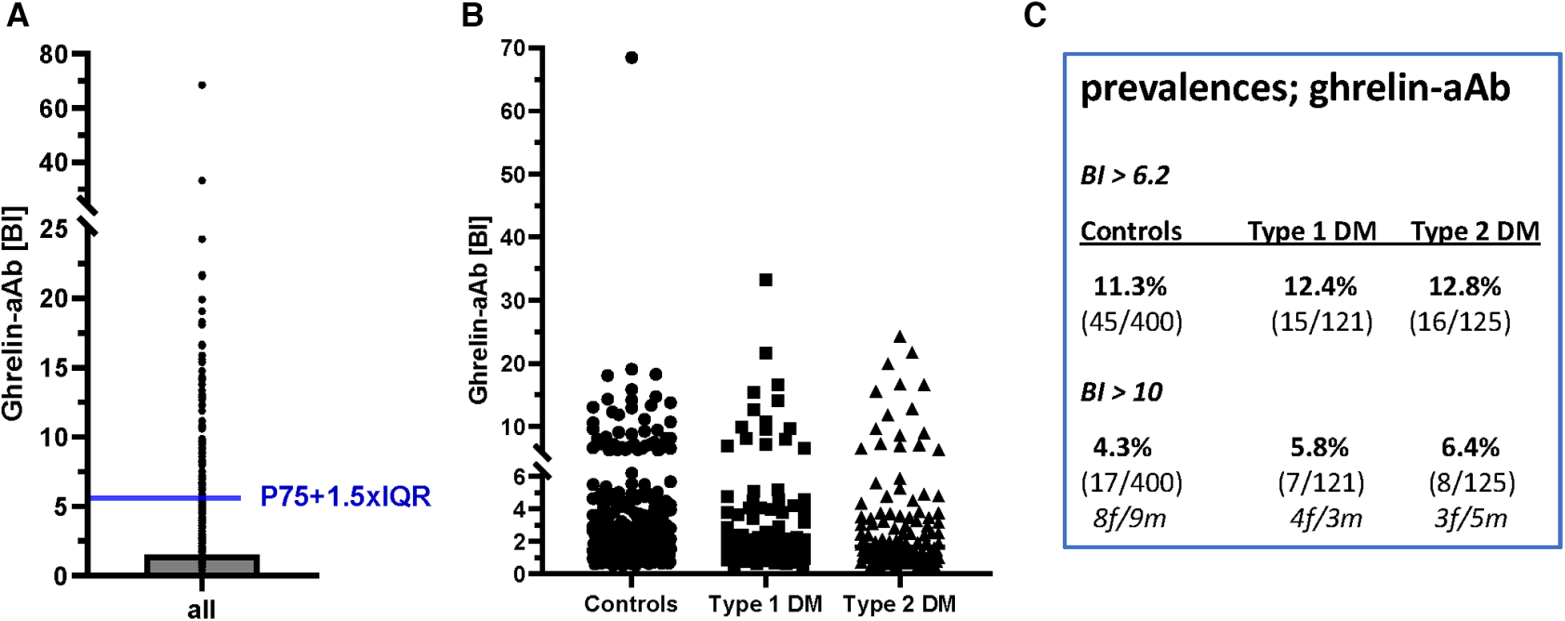
Analysis of ghrelin-aAb in healthy controls and diabetic patients. The signals from the full set of samples were converted to relative binding indices (BI). A BI of 1.0 denotes the average signal strength in the low half of all samples analyzed. (**A**) Applying a mathematical outlier criterion (P75 + 1.5-times IQR), the threshold for positivity starts at a BI of 6.2. (**B**) Applying this threshold, or a higher threshold of BI > 10 for highly positive samples, the prevalence for ghrelin-aAb is similar in healthy subjects and patients with T1DM or T2DM. (**C**) A tabular overview on the number of ghrelin-aAb positive samples indicates no significant differences between controls and patients, or men and women.

Next, three human cohorts of serum samples were analyzed with the newly generated ghrelin-aAb assay, comprising healthy men and women (*n* = 400, 50% female), and patients with T1DM (*n* = 121, 50% female) or T2DM (*n* = 124, 47% female). Comparing the signal strengths obtained, a highly skewed distribution was observed with several samples displaying strong responses. Signals were normalized according to the average background noise, i.e., the mean of the lower 50% of samples analyzed, which received a binding index (BI) of 1.0. After converting all signals to BI values, the threshold value for separating negative from positive samples was calculated by a mathematical outlier criterion (Figure 4A). Applying this criterion (BI = 6.2), the prevalence in the group of healthy subjects and patients with T1DM or T2DM was similar, i.e., 11.3%–12.8% of the samples analyzed were positive for ghrelin-aAb (Figure 4B). Choosing a higher threshold for very positive samples exceeding a BI > 10, again no difference in prevalence was observed (Figure 4C). The percentage of females among the ghrelin-aAb positive samples was 55% in healthy subjects, 60% in T1DM, and 50% in T2DM.

### 3.5 Comparison of ghrelin-aAb signals from serum and purified immunoglobulins

Finally, five different ghrelin-aAb-positive serum samples were selected and their Ig prepared. The measurements were conducted with the original serum samples along with the isolated Ig preparations. The order of positive samples (as % of max) was the same between the serum samples and the isolated Ig (Table 1), indicating that the signals obtained are due to the Ig-mediated precipitation of the SEAP-ghrelin reporter fusion protein, and likely not from other Ig-unrelated components that may be present in human serum samples, supporting the analytical quality of the ghrelin-aAb assay.

**Table 1:** Comparison of crude serum and isolated Ig preparations in the ghrelin-aAb assay.

Sample	Ghrelin-aAb in serum (RLU)	Relative ghrelin-aAb in serum (% of max)	Ghrelin-aAb in isolated Ig (RLU)	Relative ghrelin-aAb in Ig (% of max)
1	84,739	27	64,052	30
2	30,270	10	9,865	5
3	314,813	100	209,527	100
4	9,069	3	1,957	1
5	70,409	22	43,563	21

## 4 Discussion

In this study, we present the establishment and first characterization of a novel assay capable of detecting and quantifying ghrelin-aAb in human serum using a fusion protein precipitation method. The analysis of aAb to endocrine signals, receptors or transporters constitutes a specific challenge, as these biomolecules usually undergo intensive posttranslational modifications, including proteolytic processing, glycation or even acylation as in case of ghrelin (24). For this reason, screening assays using short linear peptides or recombinant prokaryotic protein often fail to provide conclusive signals, and biological tests pose specific requirements on the sample and preparation of detector molecules (25). The analytical assay presented here takes advantage of full-length ghrelin expressed via the secretory pathway in a human cell line, thereby yielding a high probability of correct folding and modifications. However, on the one hand, as a fusion protein with an enzymatically active reporter molecule, the N-terminus of the antigen is not as well accessible to ghrelin-aAb as that of the natural hormone itself. On the other hand, the use of a fusion protein of ghrelin with SEAP enables a straightforward assay that does not depend on a rather unspecific detection of antibodies recognizing the Fc part of all possible antibodies in a given test tube, thereby reducing noise and yielding higher and more specific signals (26). This notion is supported by the consistent results with the commercial anti-ghrelin antibody, verifying the basic test principle, and enabling a universal comparison of different ghrelin-aAb tests by using the same commercial source of antibodies as a positive calibrator. Such standards along with certified reference materials offer the chance to directly standardize different detection and quantification methods to enable cross-method validation experiments (27).

Concerning ghrelin-aAb characteristics, serum turned out to be the most suitable matrix for analysis, in line with other similar analyses (28). Importantly, ghrelin-aAb signals were stable over at least 10 days at room temperature and 4°C, enabling regular collection and sending of samples from the place of clinical care to the analytical site. Some effects of the matrix in the analyses of equi-volume mixtures were observed, along with a decline of signal strength upon repeated freezing, which highlights that some more characterization and stabilization experiments are needed in case large clinical studies are to be analyzed. As the comparison of serum as matrix with plasma preparations indicated considerable differences, the analyses of large cohorts of samples need to be conducted with one specific matrix only, and a direct comparison of signal strengths from serum and plasma samples is not possible.

Our major hypothesis concerned a potentially relevant role of ghrelin-aAb in type 1 or type 2 diabetes mellitus. By analyzing a considerable number of cases along with a high number of controls, we succeeded in identifying ghrelin-aAb positive samples by using a robust and prudent criterion for positivity. However, the results indicated a similar prevalence of ghrelin-aAb in all three cohorts of samples, with a rather uniform prevalence of 11%–13% in the examined groups using a mathematical criterion-based threshold, or a range of 4.3%–6.4% when applying a stringent threshold of at least 10-times signal strength above control. For most autoimmune diseases, there is a sex difference in prevalence, and women are usually more frequently affected than men (29). We did not observe a similar sex difference for the occurrence of ghrelin-aAb in our cohort, with a fraction of 50%–60% of the positive individuals being female.

These results are partly in line and partly in disagreement with the published results from other studies using different technologies for the detection and quantification of ghrelin-aAb. A seminal paper described an increased presence of anti-hypothalamic autoantibodies in patients with anorexia nervosa as compared to controls, and a negative correlation of the autoantibody titers with BMI in the study group (30). Unfortunately, specific autoantigenic targets could not be differentiated or analyzed separately. A study on ghrelin-aAb in patients with rheumatoid arthritis identified positive subjects in both the group of patients and of controls, with some negative relation of free and total IgG ghrelin-aAb in the patients, potentially reflecting the effects of immunosuppressive therapy by MTX (19). A study in young subjects reported a slightly higher prevalence of ghrelin-aAb in female as compared to male adolescents, a positive correlation with the waist-hip ratio, but no relation to body fat percentage or body mass index (14). Interestingly, enjoyment of food was negatively associated with ghrelin-aAb in the female participants (14). This observation accords to experimental studies, where polyclonal anti-ghrelin antibodies were applied intravenously and caused dose-dependent inhibition of feeding and food intake in rats (31).

In humans, immunization against ghrelin had entered Phase I and IIa trials as an anti-obesity therapy (32, 33), albeit without conclusive results. The underlying notion is not perfectly in line with an enhanced stability of ghrelin-aAb as shown in an observational analysis in humans, where the isolated ghrelin-aAb displayed an increased orexigenic effect and stimulated overeating upon interventional testing in experimental mice (20). Especially this report prompted our interest and contributed to our hypothesis on a postulated role of ghrelin-aAb in diabetes, as it may associate with overweight and overeating. Yet, because of the many different and incongruent findings at hand, it becomes obvious that additional and sufficiently powered cohort studies and analyses are needed to provide a more insightful picture on the covariates and readouts that are relevant for assessing the effects of ghrelin-aAb in human subjects. To this end, the newly developed assay may be of value.

In summary, we report the development and first intensive characterization of a quantitative assay capable of detecting ghrelin-aAb in human serum samples by immunoprecipitation of a reporter-ghrelin fusion protein. Among the strengths of our study is the characterization of signal stability regarding matrix, storage, and re-freezing. By using full-length human ghrelin produced in a human cell line, directly fused with a reporter enzyme in frame, the signals detected are likely specific for natural autoimmunity to ghrelin and quantitative in nature, as similarly tested before with other antigenic fusion proteins for detection of difficult endocrine target molecules (34–36).

Among the notable limitations of our assay is the dependence on protein A-mediated precipitation, which primarily selects for immunoglobulins of the IgG class. Other types of immunoglobulins may also play a relevant role, in particular in response to acute stimuli like viral infections, that may be involved as primary stimulus for ghrelin-aAb development (15). However, testing for IgG-mediated autoimmunity appears suitable for understanding long-term effects, in particular when applied to slowly developing chronic diseases like diabetes mellitus (37). Further limitations are given by the lack of specific clinical parameters from the diabetic patients analyzed, as data safety only allowed for a minimal set of information to be shared with the commercial service provider from whom the samples were obtained. Even though, the sample size was above 100 per group, it is still a limited number, particularly in view of the 4.3%–6.4% of highly positive subjects only, necessitating further studies with more intense characterization of clinical parameters and increased size of the study groups to be compared. Given the undisputable prime role of ghrelin in control of appetite, metabolism, thermogenesis, taste sensation and even the composition of the microbiota, several more relevant hypotheses can be put forward on where and when autoimmunity to ghrelin signaling may be of clinical relevance (38–40). With the newly generated knowledge on the prerequisites and reproducibility of the quantitative immunoprecipitation assay for ghrelin-aAb, such an endeavor on testing different and sufficiently sized cohorts of controls and patients appears feasible and promising, and should be conducted using the appropriate controls, calibrators, and available reference materials.

## Data Availability

The original contributions presented in the study are included in the article/Supplementary Material, further inquiries can be directed to the corresponding author.

## References

[1] Konturek PC Konturek JW Cześnikiewicz-Guzik M Brzozowski T Sito E Konturek SJ. Neuro-hormonal control of food intake: basic mechanisms and clinical implications. J Physiol Pharmacol. (2005) 56(Suppl 6):5–25. 16340035

[2] Crespo C S Cachero A P Jiménez L P Barrios V Arilla Ferreiro E. Peptides and food intake. Front Endocrinol (Lausanne). (2014) 5:58. 10.3389/fendo.2014.00058 24795698 PMC4005944

[3] Patterson M Bloom SR Gardiner JV. Ghrelin and appetite control in humans–potential application in the treatment of obesity. Peptides. (2011) 32(11):2290–4. 10.1016/j.peptides.2011.07.021 21835215

[4] Pradhan G Samson SL Sun Y. Ghrelin: much more than a hunger hormone. Curr Opin Clin Nutr Metab Care. (2013) 16(6):619–24. 10.1097/MCO.0b013e328365b9be 24100676 PMC4049314

[5] Cornejo MP Mustafá ER Cassano D Banères JL Raingo J Perello M. The ups and downs of growth hormone secretagogue receptor signaling. Febs j. (2021) 288(24):7213–29. 10.1111/febs.15718 33460513

[6] Poher AL Tschöp MH Müller TD. Ghrelin regulation of glucose metabolism. Peptides. (2018) 100:236–42. 10.1016/j.peptides.2017.12.015 29412824 PMC5805851

[7] Tschöp M Smiley DL Heiman ML. Ghrelin induces adiposity in rodents. Nature. (2000) 407(6806):908–13. 10.1038/35038090 11057670

[8] Pöykkö SM Kellokoski E Hörkkö S Kauma H Kesäniemi YA Ukkola O. Low plasma ghrelin is associated with insulin resistance, hypertension, and the prevalence of type 2 diabetes. Diabetes. (2003) 52(10):2546–53. 10.2337/diabetes.52.10.2546 14514639

[9] Tuero C Becerril S Ezquerro S Neira G Frühbeck G Rodríguez A. Molecular and cellular mechanisms underlying the hepatoprotective role of ghrelin against NAFLD progression. J Physiol Biochem. (2023) 79(4):833–49. 10.1007/s13105-022-00933-1 36417140

[10] Ezquerro S Mocha F Frühbeck G Guzmán-Ruiz R Valentí V Mugueta C Ghrelin reduces TNF-α-induced human hepatocyte apoptosis, autophagy, and pyroptosis: role in obesity-associated NAFLD. J Clin Endocrinol Metab. (2019) 104(1):21–37. 10.1210/jc.2018-01171 30137403

[11] Anderson KC Hasan F Grammer EE Kranz S. Endogenous ghrelin levels and perception of hunger: a systematic review and meta-analysis. Adv Nutr. (2023) 14(5):1226–36. 10.1016/j.advnut.2023.07.011 37536563 PMC10509419

[12] Buraczynska M Golacki J Zaluska W. Leu72Met polymorphism in ghrelin gene: a potential risk factor for hypertension in type 2 diabetes patients. Diabetes Metab Syndr Obes. (2023) 16:557–64. 10.2147/DMSO.S393373 36883139 PMC9985889

[13] Kharbanda KK Farokhnia M Deschaine SL Bhargava R Rodriguez-Flores M Casey CA Role of the ghrelin system in alcohol use disorder and alcohol-associated liver disease: a narrative review. Alcohol Clin Exp Res. (2022) 46(12):2149–59. 10.1111/acer.14967 36316764 PMC9772086

[14] Espinoza-García AS Hunot-Alexander C Martínez-Moreno AG Vázquez-Solorzano R Porchas-Quijada M Reyes-Castillo Z. IgG antibodies reacting with ghrelin and leptin are correlated with body composition and appetitive traits in young subjects. Appetite. (2022) 168:105685. 10.1016/j.appet.2021.105685 34506856

[15] Fetissov SO Hamze Sinno M Coquerel Q Do Rego JC Coëffier M Gilbert D Emerging role of autoantibodies against appetite-regulating neuropeptides in eating disorders. Nutrition. (2008) 24(9):854–9. 10.1016/j.nut.2008.06.021 18725083

[16] Terashi M Asakawa A Harada T Ushikai M Coquerel Q Sinno MH Ghrelin reactive autoantibodies in restrictive anorexia nervosa. Nutrition. (2011) 27(4):407–13. 10.1016/j.nut.2011.01.002 21392704

[17] Fetissov SO Lucas N Legrand R. Ghrelin-Reactive immunoglobulins in conditions of altered appetite and energy balance. Front Endocrinol (Lausanne). (2017) 8:10. 10.3389/fendo.2017.00010 28191004 PMC5269453

[18] Smitka K Prochazkova P Roubalova R Dvorak J Papezova H Hill M Current aspects of the role of autoantibodies directed against appetite-regulating hormones and the gut microbiome in eating disorders. Front Endocrinol (Lausanne). (2021) 12:613983. 10.3389/fendo.2021.613983 33953692 PMC8092392

[19] Porchas-Quijada M Reyes-Castillo Z Muñoz-Valle JF Durán-Barragán S Aguilera-Cervantes V López-Espinoza A IgG anti-ghrelin immune complexes are increased in rheumatoid arthritis patients under biologic therapy and are related to clinical and metabolic markers. Front Endocrinol (Lausanne). (2019) 10:252. 10.3389/fendo.2019.00252 31057488 PMC6482250

[20] Takagi K Legrand R Asakawa A Amitani H François M Tennoune N Anti-ghrelin immunoglobulins modulate ghrelin stability and its orexigenic effect in obese mice and humans. Nat Commun. (2013) 4:2685. 10.1038/ncomms3685 24158035 PMC3826639

[21] François M Schaefer JM Bole-Feysot C Déchelotte P Verhulst FC Fetissov SO. Ghrelin-reactive immunoglobulins and anxiety, depression and stress-induced cortisol response in adolescents. The TRAILS study. Prog Neuropsychopharmacol Biol Psychiatry. (2015) 59:1–7. 10.1016/j.pnpbp.2014.12.011 25562566

[22] Sun Q Mehl S Renko K Seemann P Görlich CL Hackler J Natural autoimmunity to selenoprotein P impairs selenium transport in hashimoto’s thyroiditis. Int J Mol Sci. (2021) 22(23):13088. 10.3390/ijms222313088 PMC8658221 34884891

[23] Schuette A Moghaddam A Seemann P Duda GN Schmidmaier G Schomburg L. Treatment with recombinant human bone morphogenetic protein 7 leads to a transient induction of neutralizing autoantibodies in a subset of patients. BBA Clin. (2016) 6:100–7. 10.1016/j.bbacli.2016.08.001 27617228 PMC5007422

[24] Thomas AS Sassi M Angelini R Morgan AH Davies JS. Acylation, a conductor of ghrelin function in brain health and disease. Front Physiol. (2022) 13:831641. 10.3389/fphys.2022.831641 35845996 PMC9280358

[25] Davis TR Pierce MR Novak SX Hougland JL. Ghrelin octanoylation by ghrelin O-acyltransferase: protein acylation impacting metabolic and neuroendocrine signalling. Open Biol. (2021) 11(7):210080. 10.1098/rsob.210080 34315274 PMC8316800

[26] Burbelo PD Ji Y Iadarola MJ. Advancing luciferase-based antibody immunoassays to next-generation mix and read testing. Biosensors (Basel). (2023) 13(3):303. 10.3390/bios13030303 36979515 PMC10046223

[27] Monogioudi E Zegers I. Certified reference materials and their need for the diagnosis of autoimmune diseases. Mediterr J Rheumatol. (2019) 30(1):26–32. 10.31138/mjr.30.1.26 32185339 PMC7045914

[28] Frias MA Virzi J Batuca J Pagano S Satta N Delgado Alves J ELISA methods comparison for the detection of auto-antibodies against apolipoprotein A1. J Immunol Methods. (2019) 469:33–41. 10.1016/j.jim.2019.03.011 30926534

[29] Ngo ST Steyn FJ McCombe PA. Gender differences in autoimmune disease. Front Neuroendocrinol. (2014) 35:347–69. 10.1016/j.yfrne.2014.04.004 24793874

[30] Escelsior A Cogorno L Sukkar SG Amerio A Donini LM Bellomo M Anti-hypothalamus autoantibodies in anorexia nervosa: a possible new mechanism in neuro-physiological derangement? Eat Weight Disord. (2022) 27(7):2481–96. 10.1007/s40519-022-01388-5 35297008 PMC9556421

[31] Nakazato M Murakami N Date Y Kojima M Matsuo H Kangawa K A role for ghrelin in the central regulation of feeding. Nature. (2001) 409:194–8. 10.1038/35051587 11196643

[32] Colon-Gonzalez F Kim GW Lin JE Valentino MA Waldman SA. Obesity pharmacotherapy: what is next? Mol Aspects Med. (2013) 34:71–83. 10.1016/j.mam.2012.10.005 23103610 PMC4076900

[33] Altabas V Zjacic-Rotkvic V. Anti-ghrelin antibodies in appetite suppression: recent advances in obesity pharmacotherapy. Immunotargets Ther. (2015) 4:123–30. 10.2147/ITT.S60398 27471718 PMC4918252

[34] Minich WBDN Welsink T Schwiebert C Morgenthaler NG Köhrle J Eckstein A Autoantibodies to the IGF1 receptor in graves’ orbitopathy. J Clin Endocrinol Metab. (2013) 98(2):752–60. 10.1210/jc.2012-1771 23264397

[35] Eleftheriadou AMMS Renko K Kasim RH Schaefer JA Minich WB Schomburg L. Re-visiting autoimmunity to sodium-iodide symporter and pendrin in thyroid disease. Eur J Endocrinol. (2020) 183(6):571–80. 10.1530/EJE-20-0566 33055303

[36] Minich WB Abel BS Schwiebert C Welsink T Seemann P Brown RJ A novel *in vitro* assay correlates insulin receptor autoantibodies with fasting insulin in type B insulin resistance. J Clin Endocrinol Metab. (2023) 108(9):2324–9. 10.1210/clinem/dgad125 36869714 PMC10438904

[37] Kelepouris E St Peter W Neumiller JJ Wright EE. Optimizing multidisciplinary care of patients with chronic kidney disease and type 2 diabetes Mellitus. Diabetes Ther. (2023) 14(7):1111–36. 10.1007/s13300-023-01416-2 37209236 PMC10241769

[38] Müller TD Nogueiras R Andermann ML Andrews ZB Anker SD Argente J Ghrelin. Mol Metab. (2015) 4(6):437–60. 10.1016/j.molmet.2015.03.005 26042199 PMC4443295

[39] Richards P Thornberry NA Pinto S. The gut-brain axis: identifying new therapeutic approaches for type 2 diabetes, obesity, and related disorders. Mol Metab. (2021) 46:101175. 10.1016/j.molmet.2021.101175 33548501 PMC8085592

[40] Gajewska A Strzelecki D Gawlik-Kotelnicka O. Ghrelin as a biomarker of “immunometabolic depression” and its connection with dysbiosis. Nutrients. (2023) 15(18):3960. 10.3390/nu15183960 PMC10537261 37764744

